# Correction: *Optical coherence tomography (OCT) in unconscious and systemically unwell patients using a mobile OCT device: a pilot study*

**DOI:** 10.1136/bmjopen-2019-030882corr1

**Published:** 2020-02-04

Liu X, Kale AU, Capewell N, *et al*. Optical coherence tomography (OCT) in unconscious and systemically unwell patients using a mobile OCT device: a pilot study. *BMJ Open* 2019;9:e030882. doi: 10.1136/bmjopen-2019-030882

This article was previously published with wrong licence. The correct licence for the paper is CC-BY.

