# Supplementary material for: Optical coherence tomography (OCT) in unconscious and systemically unwell patients using a mobile OCT device: a pilot study
Source: BMJ Open. 2019 Nov 7;9(11):e030882. doi: 10.1136/bmjopen-2019-030882 (PMC6858135; doi:10.1136/bmjopen-2019-030882)
Supplement: Author's manuscript [file bmjopen-2019-030882.draft_revisions.pdf]

BMJ Open is committed to open peer review. As part of this commitment we make the peer review history of every article we publish publicly available.

When an article is published we post the peer reviewers' comments and the authors' responses online. We also post the versions of the paper that were used during peer review. These are the versions that the peer review comments apply to.

The versions of the paper that follow are the versions that were submitted during the peer review process. They are not the versions of record or the final published versions. They should not be cited or distributed as the published version of this manuscript.

BMJ Open is an open access journal and the full, final, typeset and author-corrected version of record of the manuscript is available on our site with no access controls, subscription charges or pay-per-view fees (http://bmjopen.bmj.com).

If you have any questions on BMJ Open's open peer review process please email info.bmjopen@bmj.com

BMJ Open

Retinal Imaging on Critical Care: Optical Coherence Tomography (OCT) in Unconscious and Systemically Unwell Patients Using a Mobile OCT Device

Journal:	*BMJ Open*
Manuscript ID	bmjopen-2019-030882
Article Type:	Research
Date Submitted by the Author:	14-Apr-2019
Complete List of Authors:	Liu, Xiaoxuan; University Hospitals Birmingham NHS Foundation Trust, Ophthalmology Department; University of Birmingham College of Medical and Dental Sciences, Institute of Inflammation & Ageing<br>Kale, Aditya; University of Birmingham College of Medical and Dental Sciences, Institute of Inflammation & Ageing<br>Capewell, Nicholas; University Hospitals Birmingham NHS Foundation Trust, Ophthalmology Department<br>Talbot, Nicholas; University Hospitals Birmingham NHS Foundation Trust, Ophthalmology Department<br>Ahmed, Sumiya; University Hospitals Birmingham NHS Foundation Trust, Ophthalmology Department<br>Keane, Pearse; Moorfields Eye Hospital NHS Foundation Trust, NIHR Biomedical Research Centre for Ophthalmology<br>Mollan, Susan; University Hospitals Birmingham NHS Foundation Trust, Ophthalmology Department; Institute of Translational Medicine, Centre for Rare Diseases<br>belli, antonio; University of Birmingham College of Medical and Dental Sciences; University Hospitals Birmingham NHS Foundation Trust, Department of Critical Care Medicine<br>Blanch, Richard; University Hospitals Birmingham NHS Foundation Trust, Ophthalmology Department; Royal Centre for Defence Medicine, Academic Department of Military Surgery and Trauma<br>Veenith, Tonny; University of Birmingham College of Medical and Dental Sciences, Institute of Inflammation & Ageing; University Hospitals Birmingham NHS Foundation Trust, Department of Critical Care Medicine<br>Denniston, Alastair; University Hospitals Birmingham NHS Foundation Trust, Ophthalmology Department; University of Birmingham College of Medical and Dental Sciences, Institute of Inflammation & Ageing
Keywords:	optical coherence tomography, Adult intensive & critical care < ANAESTHETICS, optical coherence tomography angiography

SCHOLARONE™
Manuscripts

Retinal Imaging on Critical Care: Optical Coherence Tomography (OCT) in Unconscious and Systemically Unwell Patients Using a Mobile OCT Device

Liu X*[1,2,3], Kale A*[2], Capewell N[1], Talbot N[1], Ahmed S[1], Keane PA[3], Mollan SP[1,6], Belli A[2,4], Blanch R[1,5], Veenith T[2,4], Denniston AK[1,2,3,6]

[1]Ophthalmology Department, University Hospitals Birmingham NHS Foundation Trust, Birmingham, UK

[2]Institute of Inflammation & Ageing, College of Medical and Dental Sciences, University of Birmingham, UK

[3]NIHR Biomedical Research Centre for Ophthalmology, Moorfields Eye Hospital NHS Foundation Trust and UCL Institute of Ophthalmology, London, UK

[4]Department of Critical Care Medicine, University Hospitals Birmingham NHS Foundation Trust, Birmingham, UK

[5]Academic Department of Military Surgery and Trauma, Royal Centre for Defence Medicine, Birmingham, UK

[6]Centre for Rare Diseases, Institute of Translational Medicine, Birmingham Health Partners, UK

*Liu X and Kale A share joint first authorship.

For peer review only - http://bmjopen.bmj.com/site/about/guidelines.xhtml

Corresponding author: Prof Alastair Denniston, Academic Unit of Ophthalmology, Institute of Inflammation & Ageing, University of Birmingham, United Kingdom. Email: a.denniston@bham.ac.uk

Author Email Addresses:

- Liu X- xiaoxuan.liu@nhs.net
- Kale A- auk378@student.bham.ac.uk
- Capewell N- Nicholas.Capewell@uhb.nhs.uk
- Talbot N- nick.talbot@mac.com
- Ahmad S- sumiya_ahmed@hotmail.co.uk
- Keane PA- pearse.keane1@nhs.net
- Mollan SP- susan.mollan@uhb.nhs.uk
- Belli A- a.belli@bham.ac.uk
- Blanch R- R.J.Blanch@bham.ac.uk
- Veenith T- Tonny.Veenith@uhb.nhs.uk
- Denniston AK- a.denniston@bham.ac.uk

For peer review only

Abstract

Objectives

This study aims to evaluate the feasibility of retinal imaging in critical care using a novel mobile optical coherence tomography (OCT) device. The Heidelberg SPECTRALIS FLEX module (Heidelberg Engineering, Heidelberg, Germany) is an OCT unit with a boom arm, enabling ocular OCT assessment in less mobile patients.

Design

We undertook a prospective evaluation of the feasibility of using the SPECTRALIS FLEX for undertaking ocular OCT images in unconscious and critically ill patients.

Setting

This research was conducted in the critical care unit of a large tertiary referral unit in the UK.

Participants

13 systemically unwell patients admitted to the critical care unit, purposively sampled to enable evaluation in patients with a range of clinical states.

Outcome measures

The primary outcome was the feasibility of acquiring clinically-interpretable OCT scans on a consecutive series of patients. The standardised scanning protocol included: macula-focused OCT, OCT optic nerve head (ONH), OCT angiography (OCTA) of the macula, and ONH OCTA.

Results

For peer review only - http://bmjopen.bmj.com/site/about/guidelines.xhtml

OCT images from 13 patients were attempted. The success rates of each scan type are: OCT macula (84%), OCT ONH (76%), OCTA macula (56%) and OCTA ONH (36%). The overall mean success rate of scans per patient was 64% [95% CI 46%-81%]. Clinicians reported clinical value in 100% scans which were successfully obtained, including both ruling-in and ruling-out relevant ocular complications such as corneal thinning, macular oedema and optic disc swelling. The commonest causes of failure to achieve clinically interpretable scans were inadequately sustained OCT alignment in delirious patients and a compromised ocular surface due to corneal exposure.

Conclusions

This prospective evaluation indicates the feasibility and potential clinical value of the SPECTRALIS FLEX OCT system on the critical care unit. Portable OCT systems have the potential to bring instrument-based ophthalmic assessment to critically ill patients, enabling detection and micron-level monitoring of ocular complications.

Strengths and limitations of this study

- This study uses purposive sampling to identify a range of patients from a unique cohort, in which retinal imaging is usually not possible. This is the world's first study demonstrating the capabilities of retinal imaging in critical care, conducted in one of the largest critical care centres in Europe.
- This feasibility study uses both qualitative and quantitative data to firstly report image success, and secondly to provide information regarding image optimisation techniques for both OCT and OCTA. Obtained scans were

For peer review only

assessed by two ophthalmology specialists ensuring reliability in image interpretation and ultimately scan success rates.

- In addition to scan success and image optimisation techniques, we have included a case series investigating the strengths and limitations of using this novel OCT device in a range of patients with varied clinical states, providing an insight into the potential value of this device in critical care and ward-based patients.
- A key limitation of our study is the cohort size. We scanned 13 participants with varied success rates. Although this limits statistical power, this cohort provides a valuable insight into the value of mobile OCT devices.
- As this is a feasibility study with a novel device, there was a learning curve associated with operating the scanner. This, in conjunction with the fact that multiple operators were involved, may contribute to a degree of observer bias, although steps were taken to minimise this.

For peer review only

For peer review only - http://bmjopen.bmj.com/site/about/guidelines.xhtml

108 ## Introduction

109 Optical Coherence Tomography (OCT) is a non-invasive, light-based imaging
110 technique which enables *in vivo,* cross-sectional imaging of structures, including
111 human tissues, at high-resolution [1]. Its medical application is primarily in retinal
112 imaging where current OCT devices can now accurately resolve the individual retinal
113 layers and provide automated measures of retinal thickness estimated to the nearest
114 micron. Additionally, eye-tracking overlays enable consecutive images to be digitally
115 subtracted allowing detection of changes of even a few microns. It is thus an
116 extremely powerful tool both for diagnosis and monitoring of diseases related to the
117 retina, the optic nerve and other visualisable structures within the eye. A major
118 advance of the last couple of years has been OCT angiography in which the
119 detection of the phase-shift of erythrocytes travelling through the retinal capillaries
120 enables a detailed map of the retinal capillaries, and for the first time, a real-time
121 non-invasive map of retinal ischaemia[2].

122 The use of ophthalmic OCT and OCT angiography to detect both physiological and
123 pathological changes could be highly valuable in a critical care setting. One example
124 of its potential utility is mapping the retinal microcirculation for non-invasive
125 estimations of the microcirculation. It has been shown that retinal blood flow, as
126 measured by fluorescein angiography, exhibits prolonged retinal arterial filling time in
127 patients with lower cardiac index[3]. The same study found microvascular
128 abnormalities such as retinal microaneurysms and retinal haemorrhages (findings
129 common to diabetes and hypertension) in patients with severe sepsis. Whilst
130 fluorescein angiography has its uses, it carries significant risks such as anaphylaxis.
131 It is also not suitable for patients with renal impairment. OCT angiography on the
132 other hand is non-invasive and fast. This makes it ideal for monitoring the

For peer review only - http://bmjopen.bmj.com/site/about/guidelines.xhtml

physiological status of a patient with serial scans. Another example of where OCT is valuable is the ability to measure changes in the retinal nerve fibre layer (RNFL). There are a number of neurological conditions in which RNFL changes have been detected, such as RNFL thinning in chronic traumatic encephalopathy following traumatic brain injury and RNFL thickening seen in idiopathic intracranial hypertension[4–6]. OCT-derived measurements could provide surrogate markers of systemic disease which can guide treatment and give valuable prognostic information. So far, this has not been possible due to limitations in the design of current hardware. Most commercially available OCT platforms are static, table-top mounted machines which scan the patient in an upright, seated position. The patient places their head on a headrest in front of the OCT lens and is asked to fixate as instructed by the operator. This conventional setup is most suited for patients who are alert, compliant and, comfortable at a normal seated height and with a good degree of neck flexion. Topical mydriatics are often given to dilate the pupils for a wider aperture through which the image can be acquired, however good quality OCT scans can be acquired in undilated patients[7]. The only other commercially available alternative to the standard table-mounted OCT system is a handheld OCT (Envisu C2300, Leica Microsystems), however handheld devices are lacking in stability and are more sensitive to motion artefact and alignment issues[8,9].

The Heidelberg SPECTRALIS FLEX module (Heidelberg Engineering, Heidelberg, Germany) is a newly available modified OCT machine designed for use in a variety of clinical settings. In addition to being trolley-based (similar to a portable X-ray machine) it has a boom arm, designed to enable ocular OCT assessment in less mobile patients. The use of ocular OCT imaging as a diagnostic or monitoring tool for systemic conditions is in its infancy, and there are no studies to our knowledge

For peer review only - http://bmjopen.bmj.com/site/about/guidelines.xhtml

evaluating its role in a critical care setting. This study evaluates the feasibility of using the Heidelberg SPECTRALIS FLEX module in systemically unwell patients in a ward environment.

Methods

Study design and setting

This was a prospective evaluation of the feasibility of using the SPECTRALIS FLEX for undertaking clinically-useful ocular OCT images in unconscious and critically ill patients in a major critical care unit at University Hospitals Birmingham NHS Foundation Trust. The critical care unit is a tertiary referral unit with 68 level-three equivalent beds and one of the largest critical care units in Europe.

Approval was granted by University Hospitals Birmingham NHS Foundation Trust for this prospective evaluation as a service evaluation, the purpose being to establish feasibility of introducing the standard of care for ophthalmic assessment to the local critical care unit.

Outcome measures

The primary outcome of this evaluation was the feasibility of acquiring clinically-interpretable OCT scans on a consecutive series of patients, matched against a standard protocol. Acquired scans were reviewed by two ophthalmology specialists experienced in interpreting retinal OCT images. This was reported as percentage of patients for whom clinically-interpretable scans were obtained for each modality, and percentage of patients for whom the complete scan protocol was obtained.

Scan protocol

For peer review only - http://bmjopen.bmj.com/site/about/guidelines.xhtml

The standard scanning protocol included four scans: standard macula-focused OCT, OCT optic nerve head (ONH), OCT angiography (OCTA) of the macula, and OCTA of the ONH.

Statistical analysis

Descriptive statistics are provided with feasibility and clinical value being reported as percentages; tests of statistical significance are not relevant to this study.

OCT assessment with the SPECTRALIS FLEX

The optical system attached to the Heidelberg SPECTRALIS FLEX module is the same as the table-top platform, equipped with the ‘OCT2 system’ to provide both OCT and OCT angiography (OCTA). The major difference to the table-top system is that the acquisition lens is mounted on a flexible ‘boom’ arm which can be extended away from the main body of the module to approximately 100 cm **(Figure 1)**. The position of the flexible arm can be configured and tightened at different pivotal points along its length, thereby accommodating patients in a variety of different positions during scanning. The main body of the FLEX is mounted on wheels enabling portability of the module. The FLEX system is usually mains-powered but can operate for approximately 20 minutes on battery reserve when needed.

Figure 1. The Heidelberg SPECTRALIS FLEX module positioned around a semi-reclined examination chair and Intensive care bed, operated by research staff.

[Insert Figure 1]

For peer review only

We found several adjustments relating to position of the machine and preparation of the patient which significantly improves ease of image acquisition as described below.

Manoeuvrability and positioning

The device can be positioned comfortably at the bed-side and its extendable arm gives sufficient reach over a hospital bed (including critical care beds). Our approach to positioning the device and its operators around the bed space is shown in **Figure 1**. Operator 1 is positioned over the patient, holding the OCT camera piece in front of the patient to make small adjustments to alignment during acquisition if required. Operator 2 operates the desktop interface to lock and acquire the image and change settings in between scans using the keyboard. In an unconscious patient, having an additional operator standing behind the patient's head to hold it in position and to lift the eyelid is advantageous.

Preparation of the patient

Pupil dilation was undertaken unless clinically contraindicated. We found that pupil dilation had a much greater impact on quality of scans than with conventional OCT. The larger aperture partly compensates for problems with maintaining optical alignment which may arise from several issues including: lack of forehead and chin support, systemically unwell patients losing fixation due to fatigue, or unconscious/delirious patients not fixating at all. Ample verbal prompting, and reassurance was given during longer acquisition modalities (notably OCTA) to help the patient maintain focus. Artificial tears were given between scan acquisitions to improve the ocular surface, a common issue in unconscious patients which can impact the quality of scans.

For peer review only

For peer review only - http://bmjopen.bmj.com/site/about/guidelines.xhtml

Patient and public involvement

As this was a feasibility study evaluating novel technology, patients and the public were not involved in the design or conduct of this research.

For peer review only

For peer review only - http://bmjopen.bmj.com/site/about/guidelines.xhtml

Results

Technical feasibility

Feasibility was assessed against the completion of the full protocol of structural and vascular assessment of the retina and optic nerve (comprising OCT macula, OCT ONH, OCTA macula and OCTA ONH). OCT images from 13 patients were attempted, with at least one clinically-interpretable OCT being achieved in 92% (12/13 patients). The success rate of each scan type from the scan protocol are: OCT macula (84% successful), OCT ONH (76%), OCTA macula (56%) and OCTA ONH (36%). Completion of the full protocol was possible in 31% (4/13 patients). The mean scan protocol success rate per patient was 64% (95% confidence interval [CI] 46%-81%). **Table 1** summarises the conditions under which scans were obtained and any technical challenges.

For peer review only

Table 1. Summary of scan acquisition in all cases, success rates for completing the whole protocol and problems encountered.

Case No.	Diagnosis	Position of patient	Conscious/ Unconscious	Whole protocol achieved?	Problems encountered
1	Post-operative oesophagectomy	Sat up	Alert	Yes	No problems of note.
2	Sepsis	Supine in bed	Unconscious and intubated	No	Poor ocular surface, drifting gaze, difficult positioning due to airway.
3	Traumatic brain injury	Semi-recumbent in bed	Unconscious and intubated	No	Unable to dilate both eyes (to avoid interfering with neurological observations).
4	Toxic epidermal necrolysis (TEN)	Semi-recumbent in bed	Semi-sedated and delirious	No	Ocular surface involvement of TEN with corneal erosions and delirious patient.
5	Post-operative oesophagectomy	Semi-recumbent in bed	Alert	Yes	No problems of note.
6	Post-operative oesophagectomy	Sat up in chair	Alert	No	Patient was scanned upright in a chair with minimal head support.
7	Post-operative oesophagectomy	Semi-recumbent in bed	Alert	Yes	No problems of note.
8	Traumatic Brain Injury	Semi-recumbent in bed	Unconscious and intubated	No	Patient needed eyelids held open, difficulty achieving optimum alignment.
9	Post-operative oesophagectomy	Semi-recumbent in bed	Alert	No*	Scans were difficult to obtain even with dilation due to poor fixation and patients' lack of sleep.
10	Post-operative oesophagectomy	Sat up in chair	Alert	No*	All scans achieved without dilation.
11	Neutropenic Sepsis	Supine in bed	Unconscious	No	Patient scanned with lids held open. 3 operators were required.
12	Post-oesophagectomy	Semi-recumbent in bed	Alert	No	Patient was amblyopic in the right eye with poor fixation.
13	Post-operative oesophagectomy	Sat up in chair	Alert	No*	Patient was in pain during scans with poor fixation.

*Post-oesophagectomy scans were carried out at 24h hours post-operatively in the critical care unit. An asterisk marks where all scans were obtainable at 7 days post-operatively on a normal ward.

For peer review only

Clinical value

Clinical value was reported in 100% cases where interpretable scans were achieved. In these scans the clinical value was reported as 'ruling out' serious complications of the underlying systemic disease; in the remainder the clinical value was either diagnostic or the ability to quantitatively monitor retinal or ONH status.

Case examples

The following cases highlight the clinical value in four different contexts and demonstrate the potential clinical value of the OCT modalities available.

Case 1 - Post-operative oesophagectomy

Patient 1 was admitted for two-stage oesophagectomy for an oesophageal adenocarcinoma was scanned at day 1 post-operatively, whilst making a good recovery. At the time he was able to sit up in bed, was alert, compliant and able to maintain good fixation, enabling successful acquisition of the full scan protocol **(Figure 2)**. Serious complications including retinal ischaemia and retinal vascular occlusive disease was ruled-out based on the scans.

Figure 2. Case 1

[Insert Figure 2]

Figure Legend: The above composite image includes OCT and OCTA scans showing the retinal layers and retinal vasculature. OCT macula infrared image (A) and cross-sectional view (B). Image B is a cross section of the macula indicated by the white arrows in panels A-C and F. The bracketed area of image B indicates the retinal layers from the inner limiting membrane to the retinal pigment epithelium, below which is the choroid. Scans A and B are most commonly obtained in clinical practice and can be used to exclude retinal disease such as age-related macular degeneration. OCT optic nerve head infrared image (C) and cross-sectional scan (D). OCTA macula en face view (E) and

For peer review only

cross-sectional view (F). The area in image E marked with a border is the foveal avascular zone (a round, capillary free zone) corresponding to the area of the macula. Hyperreflective areas indicate red blood cell movement (ie. Blood flow), with hyporeflective areas indicating a lack of red blood cell movement. Cross sectional images F and H show blood flow indicated by yellow areas of the scan OCTA optic nerve head en face view (G) and cross-sectional view (H). The optic nerve head in image C corresponds to the hyporeflective area in image G. The optic nerve is highlighted by the green border in images C, G and H.

Case 2 - Sepsis

Patient 2 was admitted due to accidental paracetamol overdose with secondary renal failure, pulmonary failure and sepsis. At the time of scanning the patient was sedated and intubated. The patient was scanned in a semi-recumbent position in bed, with eyelids held open by one of the scan operators **(Figure 3)**. Additional technical challenges were (1) the poor quality of the ocular surface due to poor lid closure and inadequate lubrication resulting in corneal epitheliopathy (exposure keratopathy), and (2) patient movement resulting in an alignment artefact in the reconstructed OCTA rendition of the capillary plexi.

Figure 3. Case 2

[Insert Figure 3]

Figure Legend: Right eye OCT macula infrared (A) and cross-sectional view (B). OCTA macula en face view (C) and cross-sectional view (D). Poor ocular surface can be seen in the infrared image (A) and an alignment artefact arising from patient movement can be seen as a dark stripe on the OCTAs (C, D).

For peer review only

For peer review only - http://bmjopen.bmj.com/site/about/guidelines.xhtml

Case 3 - Traumatic brain injury

Patient 3, admitted following polytrauma and traumatic brain injury was scanned. Scans were acquired whilst the patient was intubated and sedated. The left eye (scanned eye) was fixed and dilated secondary to traumatic brain injury **(Figure 4)**. Optic nerve swelling, retinal detachment and retinal haemorrhage was successfully ruled-out in this eye using the OCT scan. The right eye was not dilated using topical mydriatics so as not to interfere with the patient's neurological observations; scan quality through the undilated pupil was poor, with acquisition of OCTA not being possible, therefore imaging in this eye was abandoned.

Figure 4. Case 3

[Insert Figure 4]

Figure Legend: OCT macula infrared image (A) and cross-sectional scan (B). OCT optic nerve head infra-red image (C) and cross-sectional scan (D). OCTA macula en face view (E) and cross-sectional view (F). OCTA optic nerve head en face view (G) and cross-sectional view (H).

Case 4 - Toxic Epidermal Necrolysis

Patient 4 who was admitted with toxic epidermal necrolysis (TEN) with 60% cutaneous involvement and corneal erosions was scanned. This patient was scanned supine; they were being weaned off sedative medication and was agitated and delirious, with a Glasgow coma scale of 10 out of 15. A macula-focused OCT was acquired, however further imaging to abandoned due to quality of scans. An additional scan - anterior segment (AS) OCT – was performed, so as to evaluate the cornea since the primary clinical concern in cases of TEN is of corneal and ocular surface disease. A good quality single-section anterior segment OCT was obtained, and serious corneal thinning was successfully ruled out. The major technical

For peer review only - http://bmjopen.bmj.com/site/about/guidelines.xhtml

challenges here were the poor ocular surface related to her disease, and the low level of patient compliance and fixation **(Figure 5).**

Figure 5. Case 4

[Insert Figure 5]

Figure Legend: OCT macula (A, B) could only be acquired using a single line scan and the quality was poor. A single line macula was obtained, but the rest of the protocol was abandoned. A single line OCT scan of the anterior segment was obtained to assess integrity of the cornea (C).

Discussion

In this prospective evaluation, we demonstrated the feasibility of using the SPECTRALIS FLEX module for undertaking clinically interpretable ocular OCT images in unconscious and critically ill patients in a critical care unit. The primary outcome of this evaluation was the feasibility of acquiring clinically-interpretable OCT. Our experience is that OCT macula was possible in 84% and OCT ONH in 76% of our cohort. OCTA was more challenging, with success rates of 56% for macula and 36% for ONH.

OCT is already recognised as a vital part of the clinical assessment for most retinal and optic nerve diseases, providing a permanent objective record and quantitative assessment of physiological and pathological status. Its clinical value is not therefore in doubt, but it is not yet clear whether this can be translated into the critical care environment.

The SPECTRALIS FLEX is a novel device. The only report of its use is in an intraoperative setting for acquiring aqueous angiographic images during phacoemulsification[10]. Handheld OCT devices are available and their use has been described in a range of patients including in children[11,12], in sedated patients[13], and in the intraoperative environment[8]. The only commercially

For peer review only

available handheld OCT device is the Envisu C2300 (Leica Microsystems, Wetzlar, Germany). This compact device weighs only 1.6 kg and is connected to a desktop system on a mobile cart via a 2-metre cable. It can acquire OCT images, but does not have OCTA capabilities. The image resolution of the SPECTRALIS FLEX module outperforms all commercially available hand-held devices since it uses the same advanced design as the bench-top system. The resolution of the FLEX system for OCTA is reported as 5.7 microns per pixel laterally and 3.9 microns per pixel axially[14]. In terms of stability and the avoidance of movement artefact, the SPECTRALIS FLEX module also outperforms hand-held systems, since operator movement is minimal. However, this does not compensate for patient movement (i.e. in the delirious patient) as seen in Case 4, resulting in the typical misalignment.

The purpose of this evaluation was to provide a ‘real-world’ assessment of the feasibility of undertaking OCT using the FLEX system. The completion of the whole protocol was only possible in 31% of patients, which reflects the challenges that need to be overcome in the critical care environment, compared to an outpatient setting. Several reasons for scan failure are common to both environments. They include: inability to maintain alignment (commonly acute confusion in critical care; dementia/learning difficulties; nystagmus and poor concentration in clinic); ocular surface disorders (commonly exposure keratopathy in critical care; any corneal pathology in clinic); media opacity (rare cause of failure in critical care; common cause in clinic due to e.g. cataracts) and extreme refractive errors. There is a learning curve with the SPECTRALIS FLEX module even among those who are experienced in using the table-top OCT systems. Our extensive experience has enabled us to refine our technical approach to improve capture, and guidance help

For peer review only

383 other users are provided as part of our protocol in the *Methods* section of this
384 manuscript.

385 In our evaluation we found that clinicians reported clinical value in all cases where
386 scans were possible, including both 'ruling-in' and 'ruling-out' relevant ocular
387 complications. These may be sight-threatening complications such as corneal
388 thinning in TEN or severe exposure keratopathy or macular oedema in diabetic eye
389 disease. Alternatively, they may reflect life-threatening CNS or systemic pathology,
390 such as retinal microvascular changes or optic disc swelling in papilloedema.
391 Although not tested with the SPECTRALIS FLEX module, standard table-top OCT
392 and OCTA has been used in a range of relevant conditions including raised
393 intracranial pressure, neurodegenerative diseases and dementia[15–18].
394 Additionally, the retinal vasculature exhibits autoregulation to systemic insults such
395 as hypoxia and has demonstrated quantifiable reductions in vessel density during
396 hypoxic provocation[19,20].

397

398 Conclusions

399 We have evaluated the feasibility of using of a novel, mobile OCT system to obtain
400 retinal scans in unconscious and critically ill patients. Portable OCT systems have
401 the potential to bring objective instrument-based ophthalmic assessment to the
402 bedside, enabling detection and micron-level monitoring of ocular complications
403 which may impact on vision or potentially serve as indicators of systemic health. This
404 prospective evaluation indicates OCT and OCT angiography is feasible on the critical
405 care unit, providing a powerful new tool for improved clinical assessment and
406 potential pathophysiological discovery in these patients.

For peer review only - http://bmjopen.bmj.com/site/about/guidelines.xhtml

Abbreviations:

- OCT- Optical Coherence Tomography
- OCTA- Optical Coherence Tomography Angiography
- ONH- Optic Nerve Head
- TEN – Toxic Epidermal Necrolysis

Declarations

Ethics approval and consent to participate

Approval was granted by University Hospitals Birmingham NHS Foundation Trust for this prospective evaluation as a service evaluation, the purpose being to establish feasibility of introducing the standard of care for ophthalmic assessment using OCT to the local critical care unit.

Availability of data and materials

The data and materials of this study can be made available upon request.

Competing interests

None.

Funding

XL, PAK and AKD receive a proportion of their funding from the Wellcome Trust, through a Health Improvement Challenge grant (200141/Z/15/Z). AKD and PAK receive a proportion of their funding from the Department of Health’s NIHR Biomedical Research Centre at Moorfields Eye Hospital and UCL Institute of Ophthalmology. TV, NT and AB receive a proortion of their funding from Queen Elizabeth Hospital Birmingham Charities.

For peer review only

Authors' contributions

AKD, TV, AB, SPM and RJB conceived the study. XL, AK, NC, SA and NT undertook data collection. XL, AK, RJB and AKD undertook data analysis. AKD, TV and RJB supervised this study. All authors participated in drafting and review of the manuscript.

Acknowledgements

We are grateful to Ali Tafreshi, Christopher Mody and Krysten Williams and the team at Heidelberg Engineering for their expertise and for providing the Heidelberg SPECTRALIS FLEX module for this study.

For peer review only - http://bmjopen.bmj.com/site/about/guidelines.xhtml

References

1 Jaffe GJ, Caprioli J. Optical coherence tomography to detect and manage retinal disease and glaucoma. Am. J. Ophthalmol. 2004;**137**:156–69. doi:10.1016/S0002-9394(03)00792-X

2 Tan ACS, Tan GS, Denniston AK, *et al.* An overview of the clinical applications of optical coherence tomography angiography. *Eye (Lond)* 2018;**32**:262–86. doi:10.1038/eye.2017.181

3 Erikson K, Liisanantti JH, Hautala N, *et al.* Retinal arterial blood flow and retinal changes in patients with sepsis: preliminary study using fluorescein angiography. *Crit Care* 2017;**21**:86. doi:10.1186/s13054-017-1676-3

4 Kathryn Bigsby BB, Bigsby K, Hasselfeld KA, *et al.* Retinal and Balance Changes Based on Concussion History: A Study of Division 1 Football Players. *Int J Phys Med Rehabil* 2014;**02**. doi:10.4172/2329-9096.1000234

5 Rebolleda G, Muñoz-Negrete FJ. Follow-up of Mild Papilledema in Idiopathic Intracranial Hypertension with Optical Coherence Tomography. *Investig Opthalmology Vis Sci* 2009;**50**:5197. doi:10.1167/iovs.08-2528

6 Mollan SP, Ali F, Hassan-Smith G, *et al.* Evolving evidence in adult idiopathic intracranial hypertension: pathophysiology and management. *J Neurol Neurosurg Psychiatry* 2016;**87**:982–92. doi:10.1136/jnnp-2015-311302

7 Tanga L, Roberti G, Oddone F, *et al.* Evaluating the effect of pupil dilation on spectral-domain optical coherence tomography measurements and their quality score. *BMC Ophthalmol* 2015;**15**. doi:10.1186/s12886-015-0168-y

8 Dayani PN, Maldonado R, Farsiu S, *et al.* Intraoperative Use of Handheld

For peer review only - http://bmjopen.bmj.com/site/about/guidelines.xhtml

Spectral Domain Optical Coherence Tomography Imaging in Macular Surgery. *Retin J Retin Vitr Dis* 2009;**29**:1457–68.

9 Sayegh SI, Nolan RM, Jung W, *et al.* Comparison of a MEMS-Based Handheld OCT Scanner With a Commercial Desktop OCT System for Retinal Evaluation Comparison of a MEMS- based handheld OCT scanner with a commercial desktop OCT system for retinal evaluation. *Tran Vis Sci Tech* 2014;**31010**. doi:10.1167/tvst.3.3.10

10 Huang AS, Camp A, Xu BY, *et al.* Aqueous Angiography: Aqueous Humor Outflow Imaging in Live Human Subjects. *Ophthalmology* 2017;**124**:1249–51. doi:10.1016/j.ophtha.2017.03.058

11 Campbell JP, Leder HA, Sepah YJ, *et al.* Wide-field Retinal Imaging in the Management of Noninfectious Posterior Uveitis. Am. J. Ophthalmol. 2012;**154**:908–911.e2. doi:10.1016/j.ajo.2012.05.019

12 Mallipatna A, Vinekar A, Jayadev C, *et al.* The use of handheld spectral domain optical coherence tomography in pediatric ophthalmology practice: Our experience of 975 infants and children. *Indian J Ophthalmol* 2015;**63**:586–93. doi:https://dx.doi.org/10.4103/0301-4738.167108

13 Avery RA, Hwang EI, Ishikawa H, *et al.* Handheld Optical Coherence Tomography During Sedation in Young Children With Optic Pathway Gliomas. *JAMA Ophthalmol* 2014;**132**:265. doi:10.1001/jamaophthalmol.2013.7649

14 Heidelberg Engineering. SPECTRALIS® OCT Angiography Module to be presented at AAO. 2016.https://www.heidelbergengineering.com/int/press-releases/spectralis-oct-angiography-module-to-be-presented-at-aao/ (accessed 22 Aug 2018).

For peer review only

For peer review only - http://bmjopen.bmj.com/site/about/guidelines.xhtml

15 Swanson JW, Aleman TS, Xu W, *et al.* Evaluation of Optical Coherence Tomography to Detect Elevated Intracranial Pressure in Children. *JAMA Ophthalmol* 2017;**135**:320. doi:10.1001/jamaophthalmol.2017.0025

16 Kaufhold F, Kadas EM, Schmidt C, *et al.* Optic nerve head quantification in idiopathic intracranial hypertension by spectral domain OCT. *PLoS One* 2012;**7**:e36965. doi:10.1371/journal.pone.0036965

17 Thomson KL, Yeo JM, Waddell B, *et al.* A systematic review and meta-analysis of retinal nerve fiber layer change in dementia, using optical coherence tomography. *Alzheimer's Dement Diagnosis, Assess Dis Monit* 2015;**1**:136–43. doi:10.1016/J.DADM.2015.03.001

18 Alvarez G, Rey A, Sanchez-Dalmau FB, *et al.* Optical coherence tomography findings in spinocerebellar ataxia-3. *Eye* 2013;**27**:1376–81. doi:10.1038/eye.2013.201

19 Pechauer AD, Jia Y, Liu L, *et al.* Optical Coherence Tomography Angiography of Peripapillary Retinal Blood Flow Response to Hyperoxia. *Investig Opthalmology Vis Sci* 2015;**56**:3287. doi:10.1167/iovs.15-16655

20 Xu H, Deng G, Jiang C, *et al.* Microcirculatory Responses to Hyperoxia in Macular and Peripapillary Regions. *Investig Opthalmology Vis Sci* 2016;**57**:4464. doi:10.1167/iovs.16-19603

For peer review only

For peer review only - http://bmjopen.bmj.com/site/about/guidelines.xhtml

Figure Titles and Legends:

Figure 1: The Heidelberg SPECTRALIS FLEX module positioning

Figure 2: Case 1

Figure Legend: The above composite image includes OCT and OCTA scans showing the retinal layers and retinal vasculature. OCT macula infrared image (A) and cross-sectional view (B). Image B is a cross section of the macula indicated by the white arrows in panels A-C and F. The bracketed area of image B indicates the retinal layers from the inner limiting membrane to the retinal pigment epithelium, below which is the choroid. Scans A and B are most commonly obtained in clinical practice and can be used to exclude retinal disease such as age-related macular degeneration. OCT optic nerve head infrared image (C) and cross-sectional scan (D). OCTA macula en face view (E) and cross-sectional view (F). The area in image E marked with a border is the foveal avascular zone (a round, capillary free zone) corresponding to the area of the macula. Hyperreflective areas indicate red blood cell movement (ie. Blood flow), with hyporeflective areas indicating a lack of red blood cell movement. Cross sectional images F and H show blood flow indicated by yellow areas of the scan OCTA optic nerve head en face view (G) and cross-sectional view (H). The optic nerve head in image C corresponds to the hyporeflective area in image G. The optic nerve is highlighted by the green border in images C, G and H.

Figure 3: Case 2

Figure Legend: Right eye OCT macula infrared (A) and cross-sectional view (B). OCTA macula en face view (C) and cross-sectional view (D). Poor ocular surface can be seen in the infrared image (A) and an alignment artefact arising from patient movement can be seen as a dark stripe on the OCTAs (C, D).

Figure 4: Case 3

Figure Legend: OCT macula infrared image (A) and cross-sectional scan (B). OCT optic nerve head infra-red image (C) and cross-sectional scan (D). OCTA macula en face view (E) and cross-sectional view (F). OCTA optic nerve head en face view (G) and cross-sectional view (H).

For peer review only

Figure 5. Case 4

Figure Legend: OCT macula (A, B) could only be acquired using a single line scan and the quality was poor. A single line macula was obtained, but the rest of the protocol was abandoned. A single line OCT scan of the anterior segment was obtained to assess integrity of the cornea (C).

For peer review only

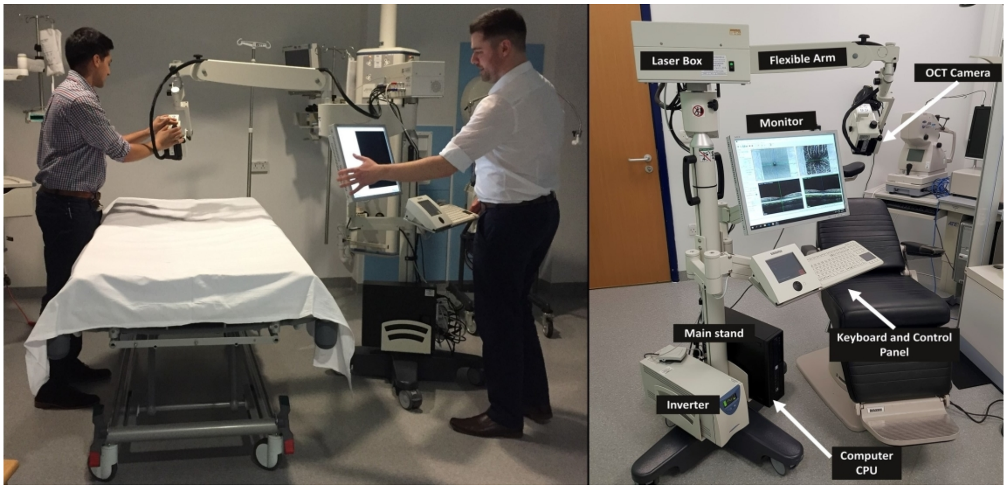

Figure 1. The Heidelberg SPECTRALIS FLEX module positioned around a semi-reclined examination chair and Intensive care bed, operated by research staff.

604x289mm (96 x 96 DPI)

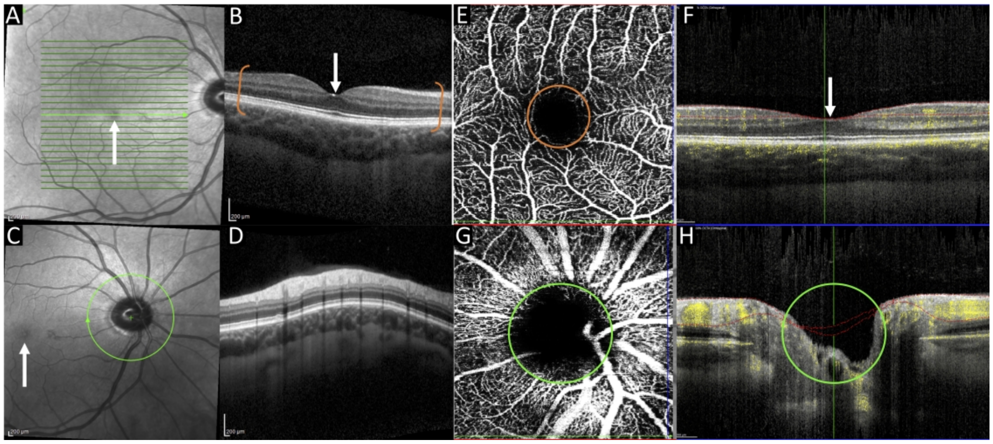

Figure Legend: The above composite image includes OCT and OCTA scans showing the retinal layers and retinal vasculature. OCT macula infrared image (A) and cross-sectional view (B). Image B is a cross section of the macula indicated by the white arrows in panels A-C and F. The bracketed area of image B indicates the retinal layers from the inner limiting membrane to the retinal pigment epithelium, below which is the choroid. Scans A and B are most commonly obtained in clinical practice and can be used to exclude retinal disease such as age-related macular degeneration. OCT optic nerve head infrared image (C) and cross-sectional scan (D). OCTA macula en face view (E) and cross-sectional view (F). The area in image E marked with a border is the foveal avascular zone (a round, capillary free zone) corresponding to the area of the macula. Hyperreflective areas indicate red blood cell movement (ie. Blood flow), with hyporeflective areas indicating a lack of red blood cell movement. Cross sectional images F and H show blood flow indicated by yellow areas of the scan OCTA optic nerve head en face view (G) and cross-sectional view (H). The optic nerve head in image C corresponds to the hyporeflective area in image G. The optic nerve is highlighted by the green border in images C, G and H.

376x170mm (300 x 300 DPI)

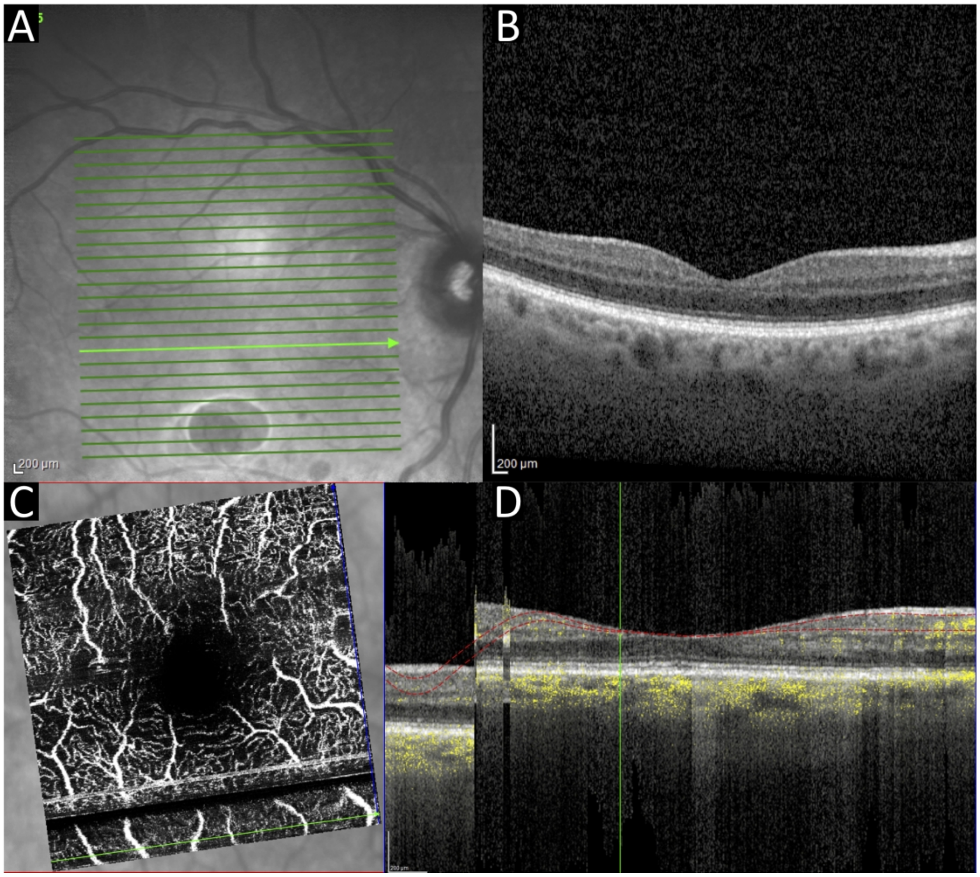

Figure Legend: Right eye OCT macula infrared (A) and cross-sectional view (B). OCTA macula en face view (C) and cross-sectional view (D). Poor ocular surface can be seen in the infrared image (A) and an alignment artefact arising from patient movement can be seen as a dark stripe on the OCTAs (C, D).

202x180mm (300 x 300 DPI)

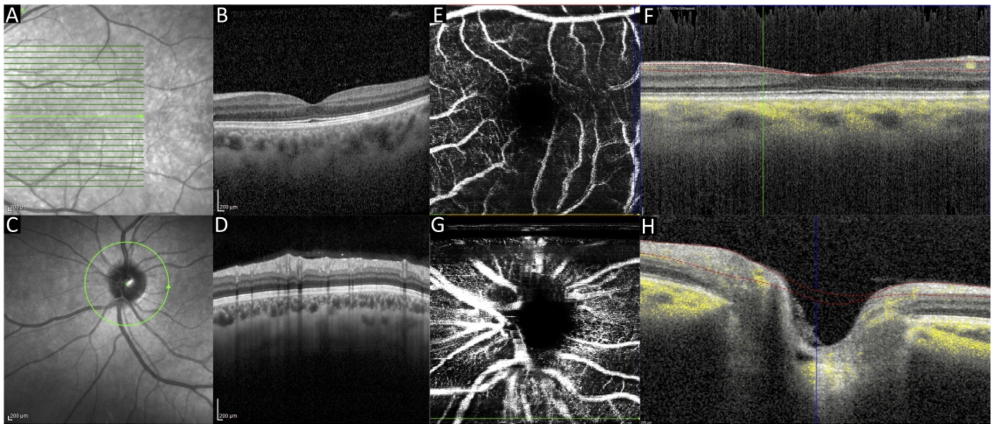

Figure Legend: OCT macula infrared image (A) and cross-sectional scan (B). OCT optic nerve head infra-red image (C) and cross-sectional scan (D). OCTA macula en face view (E) and cross-sectional view (F). OCTA optic nerve head en face view (G) and cross-sectional view (H).

422x182mm (300 x 300 DPI)

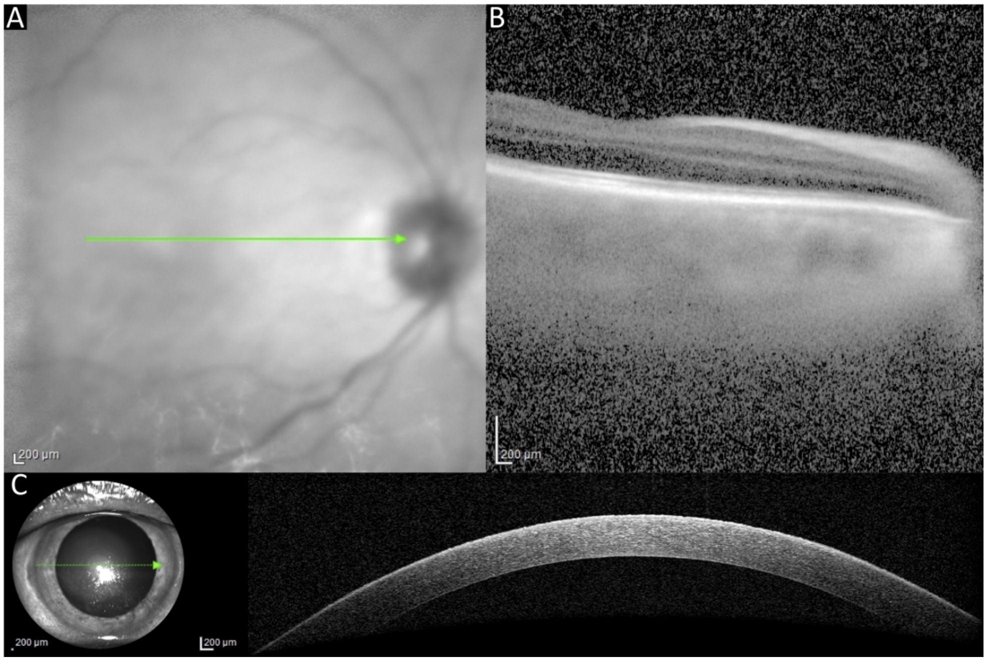

Figure Legend: OCT macula (A, B) could only be acquired using a single line scan and the quality was poor. A single line macula was obtained, but the rest of the protocol was abandoned. A single line OCT scan of the anterior segment was obtained to assess integrity of the cornea (C).

306x205mm (300 x 300 DPI)

BMJ Open

Optical Coherence Tomography (OCT) in Unconscious and Systemically Unwell Patients Using a Mobile OCT Device: A Pilot Study

Journal:	*BMJ Open*
Manuscript ID	bmjopen-2019-030882.R1
Article Type:	Original research
Date Submitted by the Author:	03-Sep-2019
Complete List of Authors:	Liu, Xiaoxuan; University Hospitals Birmingham NHS Foundation Trust, Ophthalmology Department; University of Birmingham College of Medical and Dental Sciences, Institute of Inflammation & Ageing<br>Kale, Aditya; University of Birmingham College of Medical and Dental Sciences, Institute of Inflammation & Ageing<br>Capewell, Nicholas; University Hospitals Birmingham NHS Foundation Trust, Ophthalmology Department<br>Talbot, Nicholas; University Hospitals Birmingham NHS Foundation Trust, Ophthalmology Department<br>Ahmed, Sumiya; University Hospitals Birmingham NHS Foundation Trust, Ophthalmology Department<br>Keane, Pearse; Moorfields Eye Hospital NHS Foundation Trust, NIHR Biomedical Research Centre for Ophthalmology<br>Mollan, Susan; University Hospitals Birmingham NHS Foundation Trust, Ophthalmology Department; Institute of Translational Medicine, Centre for Rare Diseases<br>belli, antonio; University of Birmingham College of Medical and Dental Sciences; University Hospitals Birmingham NHS Foundation Trust, Department of Critical Care Medicine<br>Blanch, Richard; University Hospitals Birmingham NHS Foundation Trust, Ophthalmology Department; Royal Centre for Defence Medicine, Academic Department of Military Surgery and Trauma<br>Veenith, Tonny; University of Birmingham College of Medical and Dental Sciences, Institute of Inflammation & Ageing; University Hospitals Birmingham NHS Foundation Trust, Department of Critical Care Medicine<br>Denniston, Alastair; University Hospitals Birmingham NHS Foundation Trust, Ophthalmology Department; University of Birmingham College of Medical and Dental Sciences, Institute of Inflammation & Ageing
**Primary Subject Heading**:	Ophthalmology
Secondary Subject Heading:	Intensive care
Keywords:	optical coherence tomography, Adult intensive & critical care < ANAESTHETICS, optical coherence tomography angiography

SCHOLARONE™
Manuscripts

Optical Coherence Tomography (OCT) in Unconscious and Systemically Unwell Patients Using a Mobile OCT Device: A Pilot Study

Liu X*[1,2,3], Kale AU*[2], Capewell N[1], Talbot N[1], Ahmed S[1], Keane PA[3], Mollan S[1,6], Belli A[2,4], Blanch RJ[1,5], Veenith T[2,4], Denniston AK[1,2,3,6,7]

[1]Ophthalmology Department, University Hospitals Birmingham NHS Foundation Trust, Birmingham, UK

[2]Institute of Inflammation & Ageing, College of Medical and Dental Sciences, University of Birmingham, UK

[3]NIHR Biomedical Research Centre for Ophthalmology, Moorfields Eye Hospital NHS Foundation Trust and UCL Institute of Ophthalmology, London, UK

[4]Department of Critical Care Medicine, University Hospitals Birmingham NHS Foundation Trust, Birmingham, UK

[5]Academic Department of Military Surgery and Trauma, Royal Centre for Defence Medicine, Birmingham, UK

[6]Centre for Rare Diseases, Institute of Translational Medicine, Birmingham Health Partners, UK

[7]Health Data Research UK, London, UK

*Liu X and Kale AU share joint first authorship.

Corresponding author: Prof Alastair Denniston, Academic Unit of Ophthalmology, Institute of Inflammation & Ageing, University of Birmingham, United Kingdom. Email: a.denniston@bham.ac.uk

Author Email Addresses:

For peer review only

- Liu X- xiaoxuan.liu@nhs.net
- Kale AU- aditya.u.kale@gmail.com
- Capewell N- Nicholas.Capewell@uhb.nhs.uk
- Talbot N- nick.talbot@mac.com
- Ahmad S- sumiya_ahmed@hotmail.co.uk
- Keane PA- pearse.keane1@nhs.net
- Mollan S- susan.mollan@uhb.nhs.uk
- Belli A- a.belli@bham.ac.uk
- Blanch RJ- R.J.Blanch@bham.ac.uk
- Veenith T- Tonny.Veenith@uhb.nhs.uk
- Denniston AK- a.denniston@bham.ac.uk

For peer review only

Abstract

Objectives

This study aims to evaluate the feasibility of retinal imaging in critical care using a novel mobile optical coherence tomography (OCT) device. The Heidelberg SPECTRALIS FLEX module (Heidelberg Engineering, Heidelberg, Germany) is an OCT unit with a boom arm, enabling ocular OCT assessment in less mobile patients.

Design

We undertook an evaluation of the feasibility of using the SPECTRALIS FLEX for undertaking ocular OCT images in unconscious and critically ill patients.

Setting

This study was conducted in the critical care unit of a large tertiary referral unit in the UK.

Participants

13 systemically unwell patients admitted to the critical care unit were purposively sampled to enable evaluation in patients with a range of clinical states.

Outcome measures

The primary outcome was the feasibility of acquiring clinically-interpretable OCT scans on a consecutive series of patients. The standardised scanning protocol included: macula-focused OCT, OCT optic nerve head (ONH), OCT angiography (OCTA) of the macula, and ONH OCTA.

Results

For peer review only

OCT images from 13 patients were attempted. The success rates of each scan type are: OCT macula (84%), OCT ONH (76%), OCTA macula (56%) and OCTA ONH (36%). The overall mean success rate of scans per patient was 64% [95% CI 46%-81%]. Clinicians reported clinical value in 100% scans which were successfully obtained, including both ruling-in and ruling-out relevant ocular complications such as corneal thinning, macular oedema and optic disc swelling. The commonest causes of failure to achieve clinically interpretable scans were inadequately sustained OCT alignment in delirious patients and a compromised ocular surface due to corneal exposure.

Conclusions

This prospective evaluation indicates the feasibility and potential clinical value of the SPECTRALIS FLEX OCT system on the critical care unit. Portable OCT systems have the potential to bring instrument-based ophthalmic assessment to critically ill patients, enabling detection and micron-level monitoring of ocular complications.

Strengths and limitations of this study

- This is the first study demonstrating the capabilities of the OCT FLEX device in critical care, conducted in one of the largest critical care centres in Europe.
- This feasibility study uses both qualitative and quantitative data to firstly report image success, and secondly to provide information regarding image optimisation techniques for both OCT and OCTA.
- In addition to scan success and image optimisation techniques, we have included a case series investigating the strengths and limitations of using this novel OCT device in a range of patients with varied clinical states, providing

For peer review only - http://bmjopen.bmj.com/site/about/guidelines.xhtml

83 an insight into the potential value of this device in critical care and ward-based
84 patients.

85 • A key limitation of our study is the cohort size of 13 participants and although
86 this limits statistical power, this cohort provides a valuable insight into the
87 value of mobile OCT devices.

88

For peer review only

For peer review only - http://bmjopen.bmj.com/site/about/guidelines.xhtml

Introduction

Optical Coherence Tomography (OCT) is a non-invasive, light-based imaging technique which enables *in vivo,* cross-sectional imaging of structures, including human tissues, at high-resolution [1]. Its medical application is primarily in retinal imaging where current OCT devices can now accurately resolve the individual retinal layers and provide automated measures of retinal thickness estimated to the nearest micron. Additionally, eye-tracking overlays enable consecutive images to be digitally subtracted allowing detection of changes of even a few microns. It is thus an extremely powerful tool both for diagnosis and monitoring of diseases related to the retina, the optic nerve and other visualisable structures within the eye. A major advance of the last couple of years has been OCT angiography in which the detection of the phase-shift of erythrocytes travelling through the retinal capillaries enables a detailed map of the retinal capillaries, and for the first time, a real-time non-invasive map of retinal ischaemia[2].

The use of ophthalmic OCT and OCT angiography to detect both physiological and pathological changes could be highly valuable in a critical care setting. One example of its potential utility is mapping the retinal microcirculation for non-invasive estimations of the microcirculation. It has been shown that retinal blood flow, as measured by fluorescein angiography, exhibits prolonged retinal arterial filling time in patients with lower cardiac index[3]. The same study found microvascular abnormalities such as retinal microaneurysms and retinal haemorrhages (findings common to diabetes and hypertension) in patients with severe sepsis. Whilst fluorescein angiography has its uses, it carries significant risks such as anaphylaxis. It is also not suitable for patients with renal impairment. OCT angiography on the other hand is non-invasive and fast. This makes it ideal for monitoring the

For peer review only

For peer review only - http://bmjopen.bmj.com/site/about/guidelines.xhtml

physiological status of a patient with serial scans. Another example of where OCT is valuable is the ability to measure changes in the retinal nerve fibre layer (RNFL). There are a number of neurological conditions in which RNFL changes have been detected, such as RNFL thinning in chronic traumatic encephalopathy following traumatic brain injury and RNFL thickening seen in idiopathic intracranial hypertension[4–6]. OCT-derived measurements could provide surrogate markers of systemic disease which can guide treatment and give valuable prognostic information. So far, this has not been possible due to limitations in the design of current hardware. Most commercially available OCT platforms are static, table-top mounted machines which scan the patient in an upright, seated position. The patient places their head on a headrest in front of the OCT lens and is asked to fixate as instructed by the operator. This conventional setup is most suited for patients who are alert, compliant and, comfortable at a normal seated height and with a good degree of neck flexion. Topical mydriatics are often given to dilate the pupils for a wider aperture through which the image can be acquired, however good quality OCT scans can be acquired in undilated patients[7]. The only other commercially available alternative to the standard table-mounted OCT system is a handheld OCT (Envisu C2300, Leica Microsystems), however handheld devices are lacking in stability and are more sensitive to motion artefact and alignment issues[8,9].

The Heidelberg SPECTRALIS FLEX module (Heidelberg Engineering, Heidelberg, Germany) is a newly available modified OCT machine designed for use in a variety of clinical settings. In addition to being trolley-based (similar to a portable X-ray machine) it has a boom arm, designed to enable ocular OCT assessment in less mobile patients. The use of ocular OCT imaging as a diagnostic or monitoring tool for systemic conditions is in its infancy, and there are no studies to our knowledge

For peer review only

evaluating its role in a critical care setting. This study evaluates the feasibility of using the Heidelberg SPECTRALIS FLEX module in systemically unwell patients in a ward environment.

Methods

Study design and setting

This was a cross-sectional evaluation of the feasibility of using the SPECTRALIS FLEX for undertaking clinically useful ocular OCT images in unconscious and critically ill patients in a major critical care unit at University Hospitals Birmingham NHS Foundation Trust. The critical care unit is a tertiary referral unit with 68 level-three equivalent beds and one of the largest critical care units in Europe.

Approval was granted by University Hospitals Birmingham NHS Foundation Trust for this prospective evaluation as a service evaluation, the purpose being to establish feasibility of introducing the standard of care for ophthalmic assessment to the local critical care unit. This study was carried from January to August 2018.

Outcome measures

The primary outcome of this evaluation was the feasibility of acquiring clinically-interpretable OCT scans on a consecutive series of patients, matched against a standard protocol. Acquired scans were reviewed by two ophthalmology specialists experienced in interpreting retinal OCT images. This was reported as percentage of patients for whom clinically-interpretable scans were obtained for each modality, and percentage of patients for whom the complete scan protocol was obtained. A 100% success rate was defined as all scans successful in both eyes.

Scan protocol

For peer review only - http://bmjopen.bmj.com/site/about/guidelines.xhtml

The standard scanning protocol included four scans: standard macula-focused OCT, OCT optic nerve head (ONH), OCT angiography (OCTA) of the macula, and OCTA of the ONH. This protocol was attempted for both eyes of each patient. The OCT scans used were the Heidelberg Engineering preset ‘Fast Macula’ and optic nerve head (retinal nerve fibre layer) scans. The Fast Macula scan is composed of 25 B scans at ART 9 (9 images averaged) with over an area of 5.7mm^2. The ONH scan is composed of a single B scan ring around the optic nerve head, with an ART level of 100. The OCTA scans were composed of 512 B scans, totalling an area of 2.8mm^2, with an ART level of 5.

Statistical analysis

Descriptive statistics are provided with feasibility and clinical value being reported as percentages; tests of statistical significance are not relevant to this study.

OCT assessment with the SPECTRALIS FLEX

The optical system attached to the Heidelberg SPECTRALIS FLEX module is the same as the table-top platform, equipped with the ‘OCT2 system’ to provide both OCT and OCT angiography (OCTA). The major difference to the table-top system is that the acquisition lens is mounted on a flexible ‘boom’ arm which can be extended away from the main body of the module to approximately 100 cm **(Figure 1)**. The position of the flexible arm can be configured and tightened at different pivotal points along its length, thereby accommodating patients in a variety of different positions during scanning. The main body of the FLEX is mounted on wheels enabling portability of the module. The FLEX system is usually mains-powered but can operate for approximately 20 minutes on battery reserve when needed.

For peer review only

Figure 1. The Heidelberg SPECTRALIS FLEX module positioned around a semi-reclined examination chair and Intensive care bed, operated by clinical staff.

[Insert Figure 1]

We found several adjustments relating to position of the machine and preparation of the patient which significantly improves ease of image acquisition as described below. Operators were already proficient in operating table-top OCT scanners, however training over approximately 2 hours was conducted to allow the staff to familiarise themselves with operation and manoeuvring of the FLEX module.

Manoeuvrability and positioning

The device can be positioned comfortably at the bed-side and its extendable arm gives sufficient reach over a hospital bed (including critical care beds). Our approach to positioning the device and its operators around the bed space is shown in **Figure 1**. Operator 1 is positioned over the patient, holding the OCT camera piece in front of the patient to make small adjustments to alignment during acquisition if required. Operator 2 operates the desktop interface to lock and acquire the image and change settings in between scans using the keyboard. In an unconscious patient, having an additional operator standing behind the patient's head to hold it in position and to lift the eyelid is advantageous.

Preparation of the patient

Pupil dilation was undertaken unless clinically contraindicated. We found that pupil dilation had a much greater impact on quality of scans than with conventional OCT. The larger aperture partly compensates for problems with maintaining optical alignment which may arise from several issues including: lack of forehead and chin

For peer review only - http://bmjopen.bmj.com/site/about/guidelines.xhtml

support, systemically unwell patients losing fixation due to fatigue, or unconscious/delirious patients not fixating at all. Ample verbal prompting, and reassurance was given during longer acquisition modalities (notably OCTA) to help the patient maintain focus. Artificial tears were given between scan acquisitions to improve the ocular surface, a common issue in unconscious patients which can impact the quality of scans.

Patient and public involvement

As this was a pilot study evaluating novel technology, patients and the public were not involved in the design or conduct of this study.

For peer review only

For peer review only - http://bmjopen.bmj.com/site/about/guidelines.xhtml

Results

Technical feasibility

Feasibility was assessed against the completion of the full protocol of structural and vascular assessment of the retina and optic nerve (comprising OCT macula, OCT ONH, OCTA macula and OCTA ONH). OCT images from 13 patients were attempted, with at least one clinically-interpretable OCT being achieved in 92% (12/13 patients). None of the included patients had known existing ocular pathology. The success rate of each scan type from the scan protocol are: OCT macula (84% successful), OCT ONH (76%), OCTA macula (56%) and OCTA ONH (36%). Completion of the full protocol was possible in 31% (4/13 patients). The mean scan protocol success rate per patient was 64% (95% confidence interval [CI] 46%-81%). **Table 1** summarises the conditions under which scans were obtained.

For peer review only - http://bmjopen.bmj.com/site/about/guidelines.xhtml

Table 1. Summary of scan acquisition in all cases, success rates for completing the whole protocol and problems encountered.

Case No.	Diagnosis	Patient Age	Patient sex	Position of patient	Conscious/ Unconscious	Number of scans acquired	Whole protocol achieved?	Problems encountered
1	Post-operative oesophagectomy	55	Male	Sat up	Alert	8	Yes	No problems of note.
2	Sepsis	64	Female	Supine in bed	Unconscious and intubated	3	No	Poor ocular surface, drifting gaze, difficult positioning due to airway.
3	Traumatic brain injury	33	Female	Semi-recumbent in bed	Unconscious and intubated	4	No	Unable to dilate both eyes (to avoid interfering with neurological observations).
4	Toxic epidermal necrolysis (TEN)	39	Female	Semi-recumbent in bed	Semi-sedated and delirious	0	No	Ocular surface involvement of TEN with corneal erosions and delirious patient.
5	Post-operative oesophagectomy	75	Male	Semi-recumbent in bed	Alert	8	Yes	No problems of note.
6	Post-operative oesophagectomy	84	Female	Sat up in chair	Alert	3	No	Patient was scanned upright in a chair with minimal head support.
7	Post-operative oesophagectomy	64	Female	Semi-recumbent in bed	Alert	8	Yes	No problems of note.
8	Traumatic Brain Injury	36	Male	Semi-recumbent in bed	Unconscious and intubated	3	No	Patient needed eyelids held open, difficulty achieving optimum alignment.
9	Post-operative oesophagectomy	66	Male	Semi-recumbent in bed	Alert	4	No*	Scans were difficult to obtain even with dilation due to poor fixation and patients' lack of sleep.
10	Post-operative oesophagectomy	56	Male	Sat up in chair	Alert	6	No*	All scans achieved without dilation.
11	Neutropenic Sepsis	53	Male	Supine in bed	Unconscious	8	Yes	Patient scanned with lids held open. 3 operators were required.
12	Post-operative oesophagectomy	56	Male	Semi-recumbent in bed	Alert	3	No	Patient was amblyopic in the right eye with poor fixation.
13	Post-operative oesophagectomy	58	Male	Sat up in chair	Alert	5	No*	Patient was in pain during scans with poor fixation.

*Post-oesophagectomy scans were carried out at 24 hours post-operatively in the critical care unit. An asterisk marks where all scans were obtainable at 7 days post-operatively on a normal ward.
All patients but one (case 4) had at least one successful scan.

For peer review only

Clinical value

Clinical value was reported in 100% cases where interpretable scans were achieved. In these scans the clinical value was reported as 'ruling out' serious complications of the underlying systemic disease; in the remainder the clinical value was either diagnostic or the ability to quantitatively monitor retinal or ONH status.

Case examples

The following cases highlight the clinical value in four different contexts and demonstrate the potential clinical value of the OCT modalities available.

Case 1 - Post-operative oesophagectomy

Patient 1 was admitted for two-stage oesophagectomy for an oesophageal adenocarcinoma was scanned at day 1 post-operatively, whilst making a good recovery. At the time he was able to sit up in bed, was alert, compliant and able to maintain good fixation, enabling successful acquisition of the full scan protocol **(Figure 2)**. Serious complications including retinal ischaemia and retinal vascular occlusive disease was ruled-out based on the scans.

Figure 2. Case 1

[Insert Figure 2]

Figure Legend: The above composite image includes OCT and OCTA scans showing the retinal layers and retinal vasculature. OCT macula infrared image (A) and cross-sectional view (B). Image B is a cross section of the macula indicated by the white arrows in panels A-C and F. The bracketed area of image B indicates the retinal layers from the inner limiting membrane to the retinal pigment epithelium, below which is the choroid. Scans A and B are most commonly obtained in clinical practice and can be used to exclude retinal disease such as age-related macular degeneration. OCT optic nerve head infrared image (C) and cross-sectional scan (D). OCTA macula en face view (E) and

For peer review only

cross-sectional view (F). The area in image E marked with a border is the foveal avascular zone (a round, capillary free zone) corresponding to the area of the macula. Hyperreflective areas indicate red blood cell movement (ie. Blood flow), with hyporeflective areas indicating a lack of red blood cell movement. Cross sectional images F and H show blood flow indicated by yellow areas of the scan OCTA optic nerve head en face view (G) and cross-sectional view (H). The optic nerve head in image C corresponds to the hyporeflective area in image G. The optic nerve is highlighted by the green border in images C, G and H.

Case 2 - Sepsis

Patient 2 was admitted due to accidental paracetamol overdose with secondary renal failure, pulmonary failure and sepsis. At the time of scanning the patient was sedated and intubated. The patient was scanned in a semi-recumbent position in bed, with eyelids held open by one of the scan operators **(Figure 3)**. Additional technical challenges were (1) the poor quality of the ocular surface due to poor lid closure and inadequate lubrication resulting in corneal epitheliopathy (exposure keratopathy), and (2) patient movement resulting in an alignment artefact in the reconstructed OCTA rendition of the capillary plexi.

Figure 3. Case 2

[Insert Figure 3]

Figure Legend: Right eye OCT macula infrared (A) and cross-sectional view (B). OCTA macula en face view (C) and cross-sectional view (D). Poor ocular surface can be seen in the infrared image (A) and an alignment artefact arising from patient movement can be seen as a dark stripe on the OCTAs (C, D).

For peer review only

Case 3 - Traumatic brain injury

Patient 3, admitted following polytrauma and traumatic brain injury was scanned. Scans were acquired whilst the patient was intubated and sedated. The left eye (scanned eye) was fixed and dilated secondary to traumatic brain injury **(Figure 4)**. Optic nerve swelling, retinal detachment and retinal haemorrhage was successfully ruled-out in this eye using the OCT scan. The right eye was not dilated using topical mydriatics so as not to interfere with the patient’s neurological observations; scan quality through the undilated pupil was poor, with acquisition of OCTA not being possible, therefore imaging in this eye was abandoned.

Figure 4. Case 3

[Insert Figure 4]

Figure Legend: OCT macula infrared image (A) and cross-sectional scan (B). OCT optic nerve head infra-red image (C) and cross-sectional scan (D). OCTA macula en face view (E) and cross-sectional view (F). OCTA optic nerve head en face view (G) and cross-sectional view (H).

Case 4 - Toxic Epidermal Necrolysis

Patient 4 who was admitted with toxic epidermal necrolysis (TEN) with 60% cutaneous involvement and corneal erosions was scanned. This patient was scanned supine; they were being weaned off sedative medication and was agitated and delirious, with a Glasgow coma scale of 10 out of 15. A macula-focused OCT was acquired, however further imaging to abandoned due to quality of scans. An additional scan - anterior segment (AS) OCT – was performed, so as to evaluate the cornea since the primary clinical concern in cases of TEN is of corneal and ocular surface disease. A good quality single-section anterior segment OCT was obtained, and serious corneal thinning was successfully ruled out. The major technical

For peer review only

For peer review only - http://bmjopen.bmj.com/site/about/guidelines.xhtml

challenges here were the poor ocular surface related to her disease, and the low level of patient compliance and fixation **(Figure 5).**

Figure 5. Case 4

[Insert Figure 5]

Figure Legend: OCT macula (A, B) could only be acquired using a single line scan and the quality was poor. A single line macula was obtained, but the rest of the protocol was abandoned. A single line OCT scan of the anterior segment was obtained to assess integrity of the cornea (C).

Discussion

In this prospective evaluation, we demonstrated the feasibility of using the SPECTRALIS FLEX module for undertaking clinically interpretable ocular OCT images in unconscious and critically ill patients in a critical care unit. The primary outcome of this evaluation was the feasibility of acquiring clinically-interpretable OCT. Our experience is that OCT macula was possible in 84% and OCT ONH in 76% of our cohort. OCTA was more challenging, with success rates of 56% for macula and 36% for ONH.

OCT is already recognised as a vital part of the clinical assessment for most retinal and optic nerve diseases, providing a permanent objective record and quantitative assessment of physiological and pathological status. Its clinical value is not therefore in doubt, but it is not yet clear whether this can be translated into the critical care environment.

The SPECTRALIS FLEX is a novel device. The only report of its use is in an intraoperative setting for acquiring aqueous angiographic images during phacoemulsification[10]. Handheld OCT devices are available and their use has

For peer review only

been described in a range of patients including in children[11,12], in sedated patients[13], and in the intraoperative environment[8]. The only commercially available handheld OCT device is the Envisu C2300 (Leica Microsystems, Wetzlar, Germany). This compact device weighs only 1.6 kg and is connected to a desktop system on a mobile cart via a 2-metre cable. It can acquire OCT images, but does not have OCTA capabilities. In terms of stability and the avoidance of movement artefact, the SPECTRALIS FLEX provides better stability, since operator movement is minimal. However, this does not compensate for patient movement (i.e. in the delirious patient) as seen in Case 4, resulting in the typical misalignment.

The purpose of this evaluation was to provide a 'real-world' assessment of the feasibility of undertaking OCT using the FLEX system. The completion of the whole protocol was only possible in 31% of patients, which reflects the challenges that need to be overcome in the critical care environment, compared to an outpatient setting. Several reasons for scan failure are common to both environments. They include: inability to maintain alignment (commonly acute confusion in critical care; dementia/learning difficulties; nystagmus and poor concentration in clinic); ocular surface disorders (commonly exposure keratopathy in critical care; any corneal pathology in clinic); media opacity (rare cause of failure in critical care; common cause in clinic due to e.g. cataracts) and extreme refractive errors. There are challenges associated with the SPECTRALIS FLEX module even among those who are experienced in using the table-top OCT systems. Our extensive experience has enabled us to refine our technical approach to improve capture, and guidance help other users are provided as part of our protocol in the *Methods* section of this manuscript.

For peer review only

358 Key limitations of this study include firstly, our small sample size of 13 patients limits
359 the generalisability of these results, however even with this small number we are
360 able to provide proof-of -concept as to the value of the FLEX module in a range of
361 clinical conditions. Secondly, as a new technique, there may have been a significant
362 'learning effect' as OCT-operators had to learn to overcome the challenges of
363 moving from a stable table-top device in clinic to using the mobile FLEX unit on
364 systemically unwell patients on a critical care unit. It is worth noting however that
365 there was no general trend in the success rate of scans over time. Lastly, scan
366 success was partly dependent on the patient's clinical state, and patient fatigue
367 might reduce the success of scans attempted towards the end of our protocol. This
368 may explain the lower success rate of OCTA optic nerve head scans (which are
369 relatively more demanding and time-consuming); standard OCT scans are unlikely to
370 be affected as these are fast and less demanding for patients.

371 In our evaluation we found that clinicians reported clinical value in all cases where
372 scans were possible, including both 'ruling-in' and 'ruling-out' relevant ocular
373 complications. These may be sight-threatening complications such as corneal
374 thinning in TEN or severe exposure keratopathy or macular oedema in diabetic eye
375 disease. Alternatively, they may reflect life-threatening CNS or systemic pathology,
376 such as retinal microvascular changes or optic disc swelling in papilloedema.
377 Although not tested with the SPECTRALIS FLEX module, standard table-top OCT
378 and OCTA has been used in a range of relevant conditions including raised
379 intracranial pressure, neurodegenerative diseases and dementia[14–17].
380 Additionally, the retinal vasculature exhibits autoregulation to systemic insults such
381 as hypoxia and has demonstrated quantifiable reductions in vessel density during
382 hypoxic provocation[18,19]. Further work should aim to test the reliability and clinical

For peer review only - http://bmjopen.bmj.com/site/about/guidelines.xhtml

utility of the FLEX module and compare this to both conventional table-top devices and hand-held OCT systems. Further analysis of these devices should be undertaken in the critical care setting with a longitudinal follow-up to visualise retinal changes in patients with a range of clinical conditions.

Conclusions

We have evaluated the feasibility of using of a novel, mobile OCT system to obtain retinal scans in unconscious and critically ill patients. Portable OCT systems have the potential to bring objective instrument-based ophthalmic assessment to the bedside, enabling detection and micron-level monitoring of ocular complications which may impact on vision or potentially serve as indicators of systemic health. This prospective evaluation indicates OCT and OCT angiography is feasible on the critical care unit, providing a promising foundation for the development of a new tool for improved clinical assessment and potential pathophysiological discovery in these patients.

For peer review only

For peer review only - http://bmjopen.bmj.com/site/about/guidelines.xhtml

Abbreviations:

- OCT- Optical Coherence Tomography
- OCTA- Optical Coherence Tomography Angiography
- ONH- Optic Nerve Head
- RNFL – Retinal nerve fibre layer
- TEN – Toxic Epidermal Necrolysis

For peer review only

For peer review only - http://bmjopen.bmj.com/site/about/guidelines.xhtml

Declarations

Ethics approval and consent to participate

Approval was granted by University Hospitals Birmingham NHS Foundation Trust for this prospective evaluation as a service evaluation, the purpose being to establish feasibility of introducing the standard of care for ophthalmic assessment using OCT to the local critical care unit.

Availability of data and materials

The data and materials of this study can be made available upon request.

Competing interests

None.

Funding

No funding was received for this project. XL, PAK and AKD receive a proportion of their funding from the Wellcome Trust, through a Health Improvement Challenge grant (200141/Z/15/Z). AKD and PAK receive a proportion of their funding from the Department of Health's NIHR Biomedical Research Centre at Moorfields Eye Hospital and UCL Institute of Ophthalmology. TV, NT and AB receive a proportion of their funding from Queen Elizabeth Hospital Birmingham Charities.

Authors' contributions

AKD, PAK, TV, AB, SM and RJB conceived the study. XL, AUK, NC, SA and NT undertook data collection. XL, AUK, RJB and AKD undertook data analysis. AKD, TV and RJB supervised this study. All authors participated in drafting and review of the manuscript.

For peer review only

For peer review only - http://bmjopen.bmj.com/site/about/guidelines.xhtml

Acknowledgements

We are grateful to Ali Tafreshi, Christopher Mody and Krysten Williams and the team at Heidelberg Engineering for their expertise and for providing the Heidelberg SPECTRALIS FLEX module for this study.

For peer review only

References

1 Jaffe GJ, Caprioli J. Optical coherence tomography to detect and manage retinal disease and glaucoma. Am. J. Ophthalmol. 2004;**137**:156–69. doi:10.1016/S0002-9394(03)00792-X

2 Tan ACS, Tan GS, Denniston AK, *et al.* An overview of the clinical applications of optical coherence tomography angiography. *Eye (Lond)* 2018;**32**:262–86. doi:10.1038/eye.2017.181

3 Erikson K, Liisanantti JH, Hautala N, *et al.* Retinal arterial blood flow and retinal changes in patients with sepsis: preliminary study using fluorescein angiography. *Crit Care* 2017;**21**:86. doi:10.1186/s13054-017-1676-3

4 Kathryn Bigsby BB, Bigsby K, Hasselfeld KA, *et al.* Retinal and Balance Changes Based on Concussion History: A Study of Division 1 Football Players. *Int J Phys Med Rehabil* 2014;**02**. doi:10.4172/2329-9096.1000234

5 Rebolleda G, Muñoz-Negrete FJ. Follow-up of Mild Papilledema in Idiopathic Intracranial Hypertension with Optical Coherence Tomography. *Investig Opthalmology Vis Sci* 2009;**50**:5197. doi:10.1167/iovs.08-2528

6 Mollan SP, Ali F, Hassan-Smith G, *et al.* Evolving evidence in adult idiopathic intracranial hypertension: pathophysiology and management. *J Neurol Neurosurg Psychiatry* 2016;**87**:982–92. doi:10.1136/jnnp-2015-311302

7 Tanga L, Roberti G, Oddone F, *et al.* Evaluating the effect of pupil dilation on spectral-domain optical coherence tomography measurements and their quality score. *BMC Ophthalmol* 2015;**15**. doi:10.1186/s12886-015-0168-y

8 Dayani PN, Maldonado R, Farsiu S, *et al.* Intraoperative Use of Handheld

For peer review only

For peer review only - http://bmjopen.bmj.com/site/about/guidelines.xhtml

Spectral Domain Optical Coherence Tomography Imaging in Macular Surgery. *Retin J Retin Vitr Dis* 2009;**29**:1457–68.

9 Sayegh SI, Nolan RM, Jung W, *et al.* Comparison of a MEMS-Based Handheld OCT Scanner With a Commercial Desktop OCT System for Retinal Evaluation Comparison of a MEMS- based handheld OCT scanner with a commercial desktop OCT system for retinal evaluation. *Tran Vis Sci Tech* 2014;**31010**. doi:10.1167/tvst.3.3.10

10 Huang AS, Camp A, Xu BY, *et al.* Aqueous Angiography: Aqueous Humor Outflow Imaging in Live Human Subjects. *Ophthalmology* 2017;**124**:1249–51. doi:10.1016/j.ophtha.2017.03.058

11 Campbell JP, Leder HA, Sepah YJ, *et al.* Wide-field Retinal Imaging in the Management of Noninfectious Posterior Uveitis. Am. J. Ophthalmol. 2012;**154**:908–911.e2. doi:10.1016/j.ajo.2012.05.019

12 Mallipatna A, Vinekar A, Jayadev C, *et al.* The use of handheld spectral domain optical coherence tomography in pediatric ophthalmology practice: Our experience of 975 infants and children. *Indian J Ophthalmol* 2015;**63**:586–93. doi:https://dx.doi.org/10.4103/0301-4738.167108

13 Avery RA, Hwang EI, Ishikawa H, *et al.* Handheld Optical Coherence Tomography During Sedation in Young Children With Optic Pathway Gliomas. *JAMA Ophthalmol* 2014;**132**:265. doi:10.1001/jamaophthalmol.2013.7649

14 Swanson JW, Aleman TS, Xu W, *et al.* Evaluation of Optical Coherence Tomography to Detect Elevated Intracranial Pressure in Children. *JAMA Ophthalmol* 2017;**135**:320. doi:10.1001/jamaophthalmol.2017.0025

For peer review only

15 Kaufhold F, Kadas EM, Schmidt C, *et al.* Optic nerve head quantification in idiopathic intracranial hypertension by spectral domain OCT. *PLoS One* 2012;**7**:e36965. doi:10.1371/journal.pone.0036965

16 Thomson KL, Yeo JM, Waddell B, *et al.* A systematic review and meta-analysis of retinal nerve fiber layer change in dementia, using optical coherence tomography. *Alzheimer's Dement Diagnosis, Assess Dis Monit* 2015;**1**:136–43. doi:10.1016/J.DADM.2015.03.001

17 Alvarez G, Rey A, Sanchez-Dalmau FB, *et al.* Optical coherence tomography findings in spinocerebellar ataxia-3. *Eye* 2013;**27**:1376–81. doi:10.1038/eye.2013.201

18 Pechauer AD, Jia Y, Liu L, *et al.* Optical Coherence Tomography Angiography of Peripapillary Retinal Blood Flow Response to Hyperoxia. *Investig Opthalmology Vis Sci* 2015;**56**:3287. doi:10.1167/iovs.15-16655

19 Xu H, Deng G, Jiang C, *et al.* Microcirculatory Responses to Hyperoxia in Macular and Peripapillary Regions. *Investig Opthalmology Vis Sci* 2016;**57**:4464. doi:10.1167/iovs.16-19603

For peer review only

For peer review only - http://bmjopen.bmj.com/site/about/guidelines.xhtml

495 # Figure Titles and Legends:

496 Figure 1: The Heidelberg SPECTRALIS FLEX module positioning

497 Figure 2: Case 1

498 **Figure Legend:** The above composite image includes OCT and OCTA scans showing the retinal
499 layers and retinal vasculature. OCT macula infrared image (A) and cross-sectional view (B). Image B
500 is a cross section of the macula indicated by the white arrows in panels A-C and F. The bracketed
501 area of image B indicates the retinal layers from the inner limiting membrane to the retinal pigment
502 epithelium, below which is the choroid. Scans A and B are most commonly obtained in clinical
503 practice and can be used to exclude retinal disease such as age-related macular degeneration. OCT
504 optic nerve head infrared image (C) and cross-sectional scan (D). OCTA macula en face view (E) and
505 cross-sectional view (F). The area in image E marked with a border is the foveal avascular zone (a
506 round, capillary free zone) corresponding to the area of the macula. Hyperreflective areas indicate red
507 blood cell movement (ie. Blood flow), with hyporeflective areas indicating a lack of red blood cell
508 movement. Cross sectional images F and H show blood flow indicated by yellow areas of the scan
509 OCTA optic nerve head en face view (G) and cross-sectional view (H). The optic nerve head in image
510 C corresponds to the hyporeflective area in image G. The optic nerve is highlighted by the green
511 border in images C, G and H.

512 Figure 3: Case 2

513 **Figure Legend:** Right eye OCT macula infrared (A) and cross-sectional view (B). OCTA macula en
514 face view (C) and cross-sectional view (D). Poor ocular surface can be seen in the infrared image (A)
515 and an alignment artefact arising from patient movement can be seen as a dark stripe on the OCTAs
516 (C, D).

517 Figure 4: Case 3

518 **Figure Legend:** OCT macula infrared image (A) and cross-sectional scan (B). OCT optic nerve head
519 infra-red image (C) and cross-sectional scan (D). OCTA macula en face view (E) and cross-sectional
520 view (F). OCTA optic nerve head en face view (G) and cross-sectional view (H).

For peer review only

For peer review only - http://bmjopen.bmj.com/site/about/guidelines.xhtml

Figure 5. Case 4

Figure Legend: OCT macula (A, B) could only be acquired using a single line scan and the quality was poor. A single line macula was obtained, but the rest of the protocol was abandoned. A single line OCT scan of the anterior segment was obtained to assess integrity of the cornea (C).

For peer review only

For peer review only - http://bmjopen.bmj.com/site/about/guidelines.xhtml

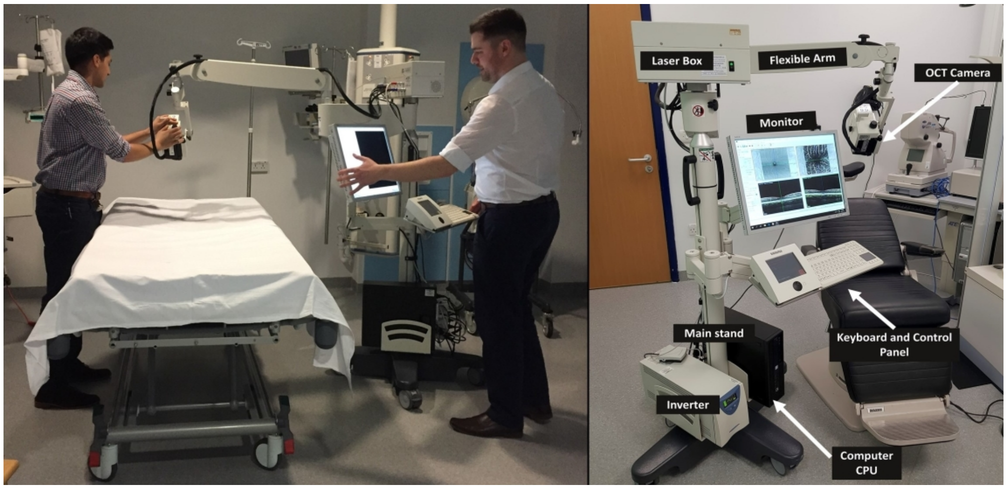

Figure 1. The Heidelberg SPECTRALIS FLEX module positioned around a semi-reclined examination chair and Intensive care bed, operated by research staff.

604x289mm (96 x 96 DPI)

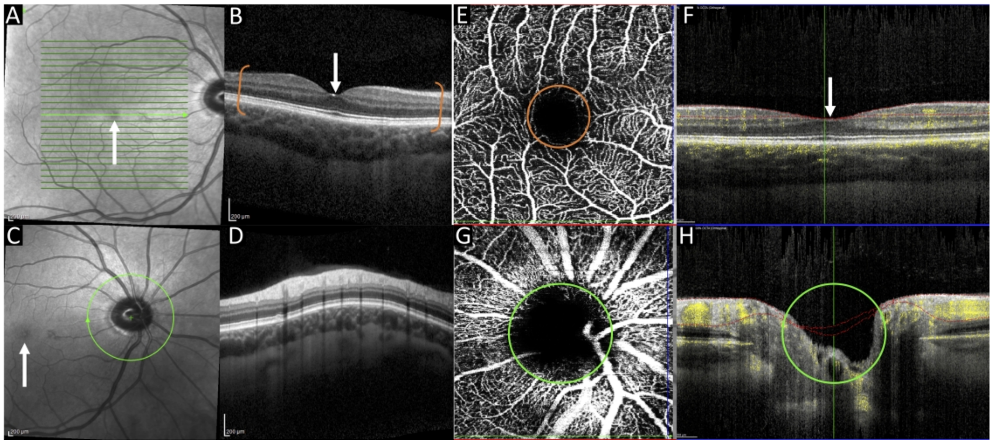

Figure Legend: The above composite image includes OCT and OCTA scans showing the retinal layers and retinal vasculature. OCT macula infrared image (A) and cross-sectional view (B). Image B is a cross section of the macula indicated by the white arrows in panels A-C and F. The bracketed area of image B indicates the retinal layers from the inner limiting membrane to the retinal pigment epithelium, below which is the choroid. Scans A and B are most commonly obtained in clinical practice and can be used to exclude retinal disease such as age-related macular degeneration. OCT optic nerve head infrared image (C) and cross-sectional scan (D). OCTA macula en face view (E) and cross-sectional view (F). The area in image E marked with a border is the foveal avascular zone (a round, capillary free zone) corresponding to the area of the macula. Hyperreflective areas indicate red blood cell movement (ie. Blood flow), with hyporeflective areas indicating a lack of red blood cell movement. Cross sectional images F and H show blood flow indicated by yellow areas of the scan OCTA optic nerve head en face view (G) and cross-sectional view (H). The optic nerve head in image C corresponds to the hyporeflective area in image G. The optic nerve is highlighted by the green border in images C, G and H.

376x170mm (300 x 300 DPI)

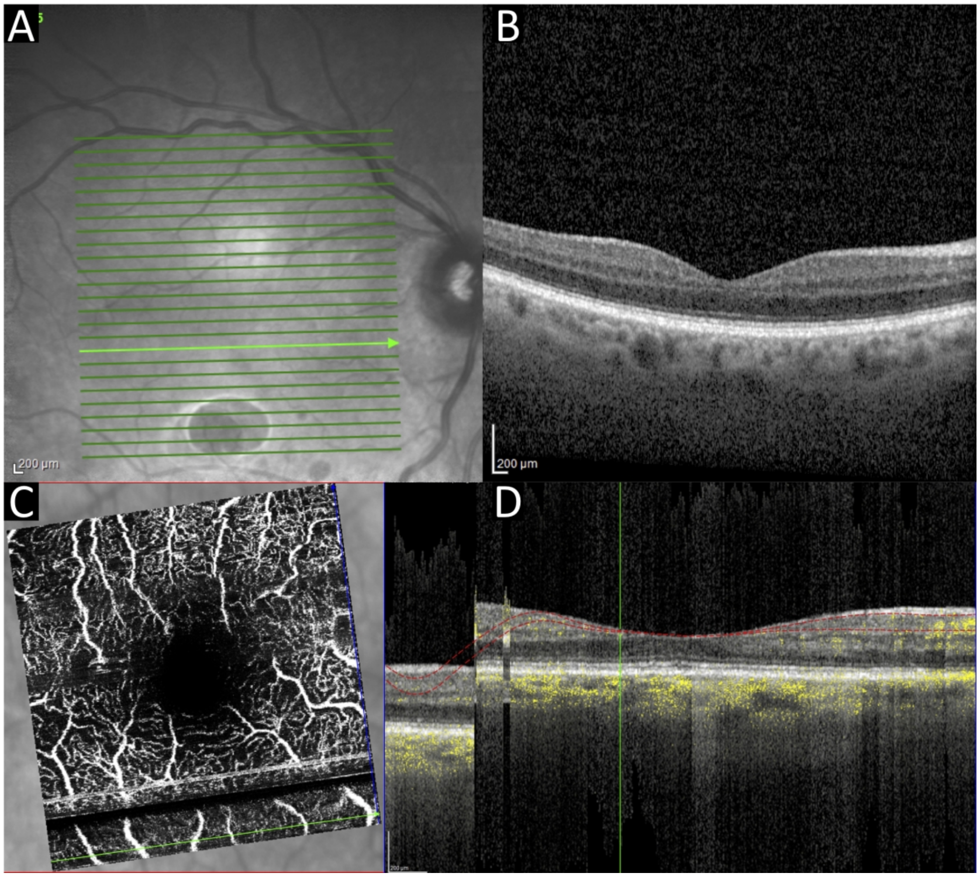

Figure Legend: Right eye OCT macula infrared (A) and cross-sectional view (B). OCTA macula en face view (C) and cross-sectional view (D). Poor ocular surface can be seen in the infrared image (A) and an alignment artefact arising from patient movement can be seen as a dark stripe on the OCTAs (C, D).

202x180mm (300 x 300 DPI)

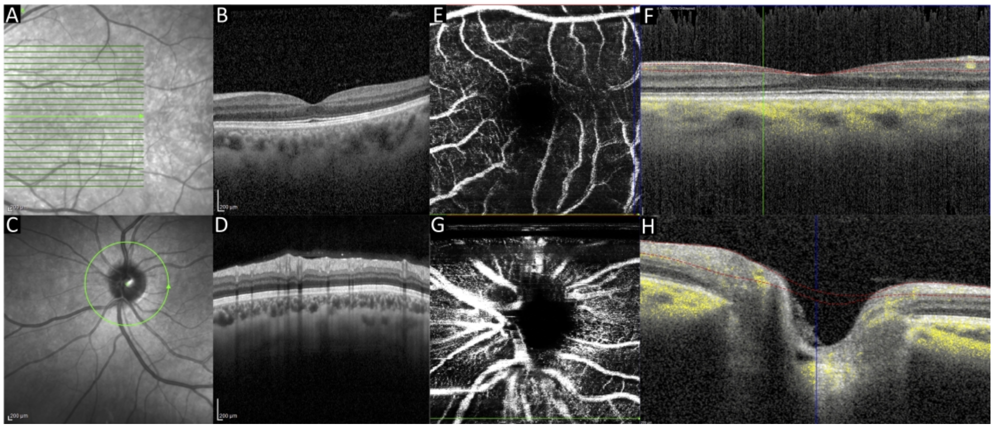

Figure Legend: OCT macula infrared image (A) and cross-sectional scan (B). OCT optic nerve head infra-red image (C) and cross-sectional scan (D). OCTA macula en face view (E) and cross-sectional view (F). OCTA optic nerve head en face view (G) and cross-sectional view (H).

422x182mm (300 x 300 DPI)

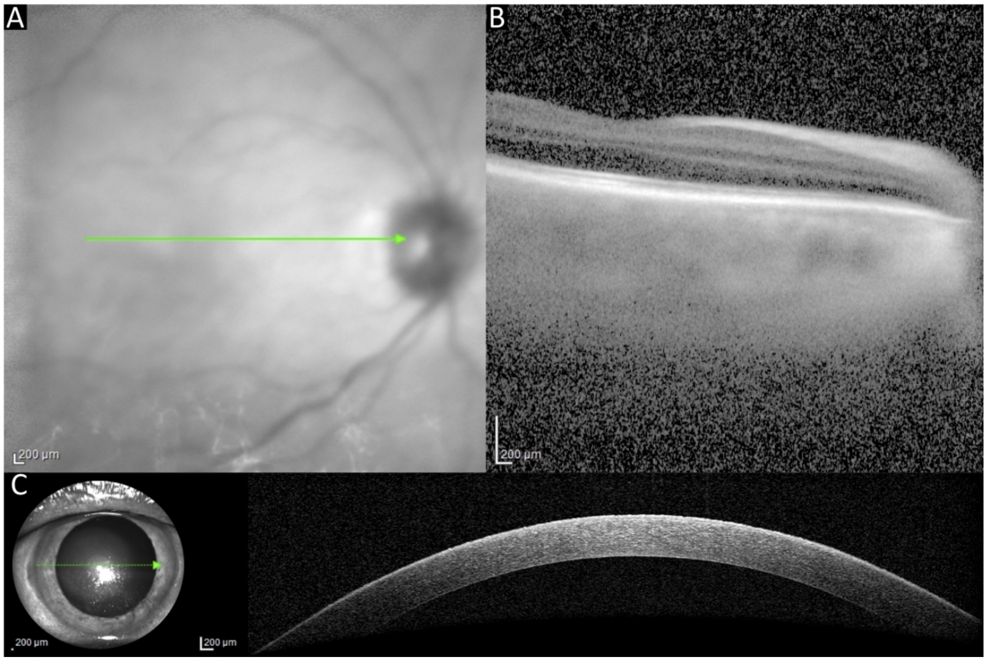

Figure Legend: OCT macula (A, B) could only be acquired using a single line scan and the quality was poor. A single line macula was obtained, but the rest of the protocol was abandoned. A single line OCT scan of the anterior segment was obtained to assess integrity of the cornea (C).

306x205mm (300 x 300 DPI)
